# Critical malnutrition thresholds and educational system constraints in Sub-Saharan Africa: implications for evidence-based nutrition policy

**DOI:** 10.3389/fnut.2026.1749392

**Published:** 2026-04-29

**Authors:** Ibtissem Gannoun, Shaykhah Aldossari, Majdi Argoubi, Khaled Mili

**Affiliations:** 1 Department of Economic Sciences and International Trade, University of Sousse, Sousse, Tunisia; 2 LARMA Research Laboratory, Sousse, Tunisia; 3 Department of Quantitative Methods, College of Business, King Faisal University, Al-Ahsa, Saudi Arabia; 4 Department of Quantitative Methods, University of Sousse, Sousse, Tunisia

**Keywords:** Child malnutrition, Educational attainment (EA), Graph Neural Networks (GNNs), Panel threshold regression (PTR) model, sub-Saharan Africa, Human capital, Threshold effects

## Abstract

**Background:**

Child malnutrition in Sub-Saharan Africa imposes substantial hidden educational costs through neurobiological mechanisms producing irreversible cognitive impairments during the “first 1,000 days”. Despite extensive micro-level evidence, macroeconomic quantification of how aggregate malnutrition constrains educational systems remains limited, particularly regarding critical threshold identification essential for geographic targeting.

**Methods:**

We integrated Hansen's Panel Threshold Regression with Temporal Graph Neural Networks analyzing 35 Sub-Saharan African countries over 1990–2024 (663 observations), enabling simultaneous identification of endogenous structural breaks and spatial interdependencies while maintaining analytical interpretability.

**Results:**

Panel Threshold Regression identified a critical threshold at 4.37% prevalence (95% CI: [3.89, 4.85])–substantially below health emergency cutoffs (WHO: 30%; UNICEF: 15%). Below this threshold (moderate regime, 22% observations, 8 countries), malnutrition was associated with a reduction in lower secondary completion of 0.31 percentage points (β_1_ = −0.312); above the threshold (critical regime, 78% observations, 27 countries), associations intensified six-fold to 1.85 percentage points (β_2_ = −1.847), consistent with exhausted compensatory mechanisms. This threshold converged with individual-level critical periods across iron (4–5%), iodine (3–5%), and protein-energy malnutrition (3–6%). Spatial analysis identified three high-vulnerability clusters (Sahel, Central Africa, East Horn) with strong within-cluster correlation (r = 0.67–0.82). Concentrating resources on critical-regime countries achieved benefit-cost ratios (15:1 to 30:1) exceeding conventional education investments (3:1 to 8:1), with 95% greater returns than uniform allocation.

**Conclusion:**

With 78% of Sub-Saharan African countries above the 4.37% threshold, achieving SDG 4 (Quality Education) requires first addressing SDG 2 (Zero Hunger). Intensive nutrition interventions–CMAM, fortified school feeding, targeted supplementation, regional coordination through ECOWAS/ECCAS/IGAD–represent prerequisite foundational investments enabling human capital accumulation.

## 1 Introduction

Child malnutrition persists as one of Sub-Saharan Africa's most formidable public health challenges, affecting approximately 31% of children under five with stunting–reaching 40–50% in Central and Sahelian regions (1, 2). Beyond immediate physiological consequences (increased infectious disease susceptibility, 45% of child deaths globally, compromised physical growth), chronic nutritional deficiencies impose substantial hidden costs across the life course through compromised brain development during the “first 1,000 days” when neurological foundations for learning are established. The World Bank estimates malnutrition costs African countries 2–16% of GDP annually, with educational impacts representing 60–70% of total economic losses through reduced labor productivity and diminished human capital formation (1, 3–5)—yet conventional assessments systematically underestimate these cognitive and educational impacts during critical developmental periods.

This nutritional-educational nexus creates a paradox particularly acute in Sub-Saharan Africa. Despite primary enrollment exceeding 75% in most countries, completion and quality remain severely compromised: average primary completion stands at 70%, Lower Secondary Completion Rate (LSCR) languishes below 45%, and several countries (Niger, Chad, CAR, Burundi) exhibit LSCR below 25% (1, 2). This underperformance cannot be attributed solely to supply-side constraints (infrastructure deficits, teacher shortages) or demand-side factors (household poverty, gender disparities), suggesting biological and cognitive barriers rooted in early nutritional deprivation may constitute a binding structural constraint overwhelming educational systems' remediation capacity. Despite extensive micro-level evidence on nutrition-cognition-education linkages, macroeconomic quantification of how aggregate malnutrition constrains national educational systems at population scale remains sparse, limiting evidence-based policy formulation.

### 1.1 Theoretical framework: integrating nutritional science, human capital economics, and educational production

Understanding how population-level malnutrition constrains educational attainment requires integrating three complementary theoretical frameworks linking chronic child malnutrition to educational failure through biological, household, and systemic mechanisms.

#### 1.1.1 Human capital theory

(6, 7) positions nutrition as a fundamental investment influencing productive capacities throughout life, emphasizing the *irreversibility* of early deficits: nutritional deprivation during critical developmental periods cannot be fully compensated by later interventions, creating path dependencies that permanently constrain human capital accumulation (8). This generates: (i) high private and social returns (3); (ii) positive externalities through reduced healthcare costs and intergenerational transmission; and (iii) poverty traps where malnutrition constrains education, limiting income and perpetuating vulnerability across generations (9).

#### 1.1.2 Educational production function

(10, 11) formalizes how inputs combine to generate outcomes, with nutritional status functioning as a *foundational input* mediating the effectiveness of all other educational investments. Malnourished children cannot fully benefit from quality teachers or facilities due to compromised cognitive capacity (12), creating multiplicative interactions where malnutrition reduces the marginal productivity of conventional inputs–implying school quality improvements yield limited returns in high-malnutrition contexts until nutritional constraints are addressed (13).

#### 1.1.3 Nutritional neuroscience

Provides the biological mechanisms. The “first 1,000 days” from conception through age two constitute a critical window of maximal brain plasticity and vulnerability (14, 15). Micronutrient deficiencies compromise brain development through multiple pathways: iron deficiency (40–60% prevalence) disrupts myelination and dopamine synthesis impairing executive functions; zinc deficiency (20–30%) compromises hippocampal neurogenesis and synaptic plasticity; iodine deficiency (15–30%) reduces thyroid hormone availability for neuronal migration; vitamin A deficiency (30–40%) increases infection burden; and protein-energy malnutrition is linked to 10–15% reduced brain volume, hippocampal atrophy, and diminished dendritic complexity (4, 15–18). These insults manifest as persistent deficits in executive functions, processing speed, and learning capacity–foundational to academic progression (14, 19, 20).

### 1.2 Transmission mechanisms: from individual deficits to system-level failure

Individual nutritional deficits aggregate into population-level educational underperformance through three interdependent pathways:

#### 1.2.1 Individual biological pathway

Early malnutrition is linked to persistent cognitive and health burdens. Children with nutritional deficits score 5–11 points lower on IQ tests and exhibit reduced working memory and impaired executive functions even after rehabilitation (14, 20), requiring more repetitions to master concepts, struggling with abstract reasoning, and showing reduced comprehension (21). Compromised immune function causes 10–15 additional school absences annually (22–24), while parasitic infections (hookworm, schistosomiasis) further compound difficulties through anemia and chronic inflammation (25).

#### 1.2.2 Household economic pathway

Malnutrition both reflects and perpetuates poverty. Food-insecure households reduce educational investments–withdrawing children for labor, delaying enrollment, or dropping out prematurely–to maintain minimal consumption (26), while healthcare costs force trade-offs between medical care and school fees (27). Parents may rationally reduce investments in malnourished children perceiving diminished returns (28), and malnourished children completing less education earn lower adult incomes, constraining their own children's nutrition and propagating intergenerational disadvantage (9, 29). Maternal malnutrition further transmits vulnerability through in utero programming (30).

#### 1.2.3 Systemic pathway

High malnutrition prevalence deteriorates classroom dynamics for all students: teachers slow instructional pace reducing curriculum coverage (31), peer effects extend cognitive impairments beyond affected individuals, and persistently low performance leads to teacher burnout and profession exit (32). Capable teachers migrate to better-nourished populations, creating spatial inequality (33), while households rationally reduce schooling demand when cognitive constraints severely limit education's achievability (34). At national scale, widespread malnutrition constrains human capital quality and economic growth (5).

### 1.3 Threshold dynamics and non-linear effects

These transmission mechanisms predict threshold dynamics rather than linear relationships. Three factors generate regime shifts: **biological thresholds**, where critical nutrient concentrations below which compensatory mechanisms fail produce irreversible damage–severe iron deficiency during infancy generates permanent dopaminergic alterations unreversible by later supplementation (15, 35); **social tipping points**, where widespread malnutrition-induced failure shifts social norms toward lower expectations, erodes teacher effort, and reduces political support (36); and **resource saturation**, where finite remedial capacity of health and education systems drops sharply once prevalence overwhelms targeted interventions (37). At population level, a critical malnutrition *prevalence threshold* thus emerges where irreversible cognitive damage overwhelms educational systems' compensatory capacity through: (i) instruction becoming ineffective when too many students face learning difficulties; (ii) exhausted household coping triggering education disinvestment; and (iii) overwhelmed system capacity.

### 1.4 Existing evidence and critical knowledge gaps

Despite substantial micro-level evidence on biological mechanisms and intervention effectiveness, the nutrition-education literature exhibits four critical gaps limiting national and regional policy guidance.

#### 1.4.1 Gap 1—macroeconomic quantification deficit

Existing studies focus on individual-level mechanisms while quantification of how aggregate prevalence constrains national *systems* remains scarce. Micro-level effect sizes do not aggregate linearly to macro-level impacts due to general equilibrium mechanisms—classroom spillovers, labor market adjustments, and fiscal feedbacks—absent from individual studies. Extrapolating from micro evaluations commits an *individualistic fallacy* (5), creating problems for policymakers requiring aggregate burden estimates and system-wide cost-effectiveness predictions.

Micro-level evidence illustrates this gap. Nutritional *quality* matters more than caloric quantity: Anderson et al. (38) find meal quality improvements yield test score gains of 0.03–0.04 SD at USD 3–5 monthly per student, with largest effects among initially malnourished children; McEwan (39) find null effects from caloric supplementation without micronutrient improvement, confirming dietary diversity drives gains. Kristjansson et al. (40)'s systematic review of 18 trials confirms school feeding increases attendance 4–6 days annually and test scores 0.02–0.05 SD, with effects 2–3 times larger among severely malnourished children. Multisectoral interventions yield larger impacts: Aryeetey et al. (41) document 15–20% stunting reductions translating into 0.12 SD cognitive gains in Ghana, with similar patterns in Ethiopia (42), Rwanda (43), and Burkina Faso (44). Targeted supplementation provides direct evidence: Lozoff et al. (35) finds iron supplementation produces executive function differences persisting 19 years post-intervention; Zimmermann (18) documents iodine programs raise population IQ 5–13 points in severely deficient regions, with sharp non-linearities–minimal benefits in mildly deficient contexts. While robustly establishing micro-level mechanisms, this literature leaves unanswered how effects aggregate at population scale, what prevalence thresholds constrain system functionality, and how patterns vary across African contexts.

#### 1.4.2 Gap 2—non-linearity and threshold neglect

Virtually all macro analyses impose linear specifications, contradicting theoretical predictions and micro-level patterns of larger effects among severely malnourished children. Linear specifications generate policy errors: underestimating impacts where threshold effects amplify damages and justifying inefficient universal interventions over geographically targeted approaches.

Household determinants literature confirms non-linearities. Filmer and Pritchett (45) establish wealth as the strongest enrollment predictor with highly non-linear effects mediated by child health; Glewwe and Jacoby (46) and Langsten (47) provide evidence that nutritional status mediates the relationship between household wealth and educational attainment in Ghana and Egypt respectively. Hanushek and Woessmann (48) document cognitive skills–not years of schooling–predict national GDP growth, with one SD increase associated with 1–2 percentage points higher annual growth, underscoring malnutrition's potential to reduce education's growth impact even when enrollment appears adequate. Hoddinott et al. (3) document benefit-cost ratios of 4:1 to 35:1 for nutrition investments, with education-related returns representing 40–60% of total benefits; Horton and Steckel (5) estimate education-related losses constitute 60–70% of malnutrition's 2–16% GDP cost. Glewwe et al. (12) and Alderman et al. (13) document strong complementarities: school quality improvements yield larger gains among adequately nourished children, while nutrition programs achieve larger income effects in better-quality school contexts—suggesting super-additive returns from integrated strategies, with unanswered questions about optimal sequencing and critical thresholds beyond which educational investments yield minimal returns.

#### 1.4.3 Gap 3—spatial interdependence oversight

Existing studies treat countries as independent observations, ignoring geographical spillovers and vulnerability clustering despite strong spatial correlation in malnutrition drivers (food insecurity, conflict, climate shocks), educational dynamics (migration, policy diffusion), and institutional factors. Neglecting spatial interdependencies produces biased estimates and overlooks regional coordination opportunities.

#### 1.4.4 Gap 4—absence of targeting frameworks

Rich mechanism evidence notwithstanding, the literature lacks *operational frameworks* for evidence-based nutrition policy targeting: which countries should prioritize intensive vs. preventive programs; at what prevalence levels do intervention modalities become cost-effective; should malnutrition be addressed *before* scaling educational investments; and where do spatial spillovers justify cross-border coordination? This gap reflects disciplinary fragmentation–nutritional science focuses on mechanisms, economics on cost-effectiveness, education research on pedagogy–that our integrated framework bridges. Addressing these four gaps requires moving beyond conventional linear panel methods to simultaneously identify critical thresholds and model spatial interdependencies–the analytical challenge motivating the research questions and methodology presented below.

### 1.5 Research questions, hypotheses, and analytical strategy

This study provides the first continental-scale empirical quantification of structural relationships between chronic child malnutrition and educational attainment in Sub-Saharan Africa, integrating insights from human capital theory, economics of education, and nutritional neuroscience to analyze how population-level malnutrition prevalence is associated with constraints on educational systems' capacity to produce skilled human capital.

Three research questions directly address identified gaps:

#### 1.5.1 RQ1—magnitude and empirical quantification

What is the macroeconomic association between chronic child malnutrition prevalence and lower secondary completion rates across Sub-Saharan Africa, controlling for GDP per capita, urbanization, education expenditure, and debt burden, and how do regime-specific marginal associations translate into benefit-cost ratios comparable to conventional education sector interventions?

#### 1.5.2 RQ2—Non-linearity and threshold identification

Does a critical malnutrition threshold (γ) exist beyond which the association with educational attainment intensifies disproportionately, reflecting regime shifts in neurobiological resilience, household coping capacity, and institutional capacity? How does this threshold align with nutritional neuroscience critical periods (iron-deficiency anemia, iodine deficiency, protein-energy malnutrition) and health-sector emergency protocols (WHO, UNICEF cutoffs), and what are the implications for differentiating preventive vs. therapeutic intervention strategies?

#### 1.5.3 RQ3—spatial structure and regional coordination

To what extent do malnutrition and educational vulnerabilities exhibit geographical clustering and cross-border spillovers, which regional clusters (Sahel, Central Africa, East Africa Horn) emerge as high-priority coordination zones, and what are the implications for regional nutrition policy frameworks through ECOWAS, ECCAS, and IGAD?

These questions correspond to three testable hypotheses: **H1—non-linear threshold effects:** a structural breakpoint should be observable in the malnutrition-education relationship, with marginal associations intensifying substantially beyond a critical prevalence threshold. **H2—spatial interdependencies:** neighboring countries' malnutrition levels should be associated with educational outcomes beyond own-country effects, validating spatial network modeling. **H3—regime-specific mechanisms:** distinct marginal associations should emerge in moderate vs. critical regimes, with household and systemic mechanisms dominating in high-malnutrition contexts.

We employ a hybrid analytical strategy combining **Panel threshold regression** **(****49****)**—identifying critical thresholds and estimating regime-specific marginal associations—with **Temporal Graph Neural Networks (GNNs)**—capturing regional spillover effects and spatial contagion dynamics. This first application of hybrid PTR-GNN methods in nutrition-education research enables simultaneous estimation of non-linear regime-specific associations (addressing Gaps 1–2) and spatial interdependencies (addressing Gap 3), providing a comprehensive evidence base for nutrition policy design.

### 1.6 Principal findings and contributions

Our empirical analysis (35 countries, 1990–2024, 663 observations) yields six principal findings:

#### 1.6.1 Critical threshold

PTR identifies $\hat{\gamma }=4.37\%$ (95% CI: [3.89, 4.85], LR test *p* < 0.001), partitioning the sample into a moderate regime (*PM* ≤ 4.37%, 22% observations, 8 countries; β_1_ = −0.312, *p* < 0.01) and a critical regime (*PM*>4.37%, 78% observations, 27 countries; β_2_ = −1.847, *p* < 0.001), with associations intensifying six-fold beyond the threshold (F = 78.34, *p* < 0.001).

#### 1.6.2 Convergence with nutritional neuroscience

The 4.37% threshold converges with individual-level critical periods across iron deficiency (4–5%), iodine deficiency (3–5%), protein-energy malnutrition reversibility (3–6%), and school feeding effectiveness amplification (5–7%), spanning micro-level neurobiology, meso-level program evaluations, and macro-level population dynamics–providing robust justification for 4.37% as an operational *policy red line*.

#### 1.6.3 Divergence from health emergency thresholds and pervasive burden

The educational impact threshold (4.37%) falls substantially below health-sector emergency cutoffs (WHO: 30%; UNICEF: 15%; Global Nutrition Report: 20%), indicating malnutrition's hidden educational costs–irreversible cognitive impairments, reduced learning capacity–emerge long before activating humanitarian protocols. Yet with 78% of countries (27/35) operating above this threshold (prevalence range: 5–49%), this is not an exceptional problem affecting outlier cases: malnutrition constitutes a pervasive structural constraint on human capital formation across the continent. Countries classified as “moderate burden” by health standards are already experiencing severe educational system impairment, justifying nutrition policy elevation to the highest levels of development strategy alongside infrastructure and governance–and preventive interventions for populations currently overlooked by health systems focused on acute cases.

#### 1.6.4 Spatial clustering

GNN analysis confirms strong regional clustering (*R*^2^ = 0.661, Moran's I *p* = 0.324) identifying three high-vulnerability clusters: **Sahel** (Niger, Chad, Mali, Burkina Faso; PM: 38.2%, LSCR: 18.4%), **Central Africa** (CAR, Chad, DRC; PM: 41.7%, LSCR: 22.1%), and **East Africa Horn** (Somalia, Djibouti, Eritrea; PM: 33.9%, LSCR: 24.8%), justifying regional coordination through ECOWAS, ECCAS, and IGAD.

#### 1.6.5 Cost-effectiveness of targeting

Concentrating 80% of a USD 1.5 billion budget on critical-regime countries achieves +2.85 percentage points LSCR gain vs. +1.46 points for uniform allocation–a 95% improvement–with benefit-cost ratios of 15:1 to 30:1 (lifetime earnings gains USD 2,500–3,000 per cohort vs. school feeding costs USD 50–80 and CMAM costs USD 150–200 per child annually) exceeding conventional education investments (teacher training: 3:1 to 8:1; infrastructure: 2:1 to 5:1).

### 1.7 Contributions and article structure

This study makes five distinct contributions: **(i) Methodological innovation**—first application of hybrid PTR-GNN methods to nutrition-education dynamics, establishing a replicable framework for threshold analysis in human development research; **(ii) Empirical quantification**—first continental-scale identification of a critical malnutrition threshold (4.37%) with regime-specific marginal associations (β_1_ = −0.31, β_2_ = −1.85) providing actionable targeting guidance; **(iii) Spatial analysis**—identification of three high-vulnerability regional clusters justifying coordinated responses through existing integration frameworks; **(iv) Policy translation**—evidence-based four-tier intervention framework differentiating preventive vs. therapeutic strategies by nutritional regime, with benefit-cost ratios (15:1 to 30:1) exceeding conventional education investments and providing operational guidance for resource-constrained national budgets; **(v) Strategic reframing**—empirical demonstration that SDG 2 (Zero Hunger) is *prerequisite* for SDG 4 (Quality Education), repositioning nutrition interventions as core educational sector investments rather than auxiliary health expenditures.

The remainder proceeds as follows. Section 2 describes methodology, data sources, and validation protocols. Section 3 presents empirical results including threshold identification, regime-specific associations, convergent validity, robustness checks, and spatial analysis. Section 4 translates findings into actionable policy recommendations including a four-tier intervention framework, regional coordination mechanisms, and SDG integration strategy. Section 5 concludes with contributions, limitations, and future research directions.

## 2 Materials and methods

This section operationalizes our analytical strategy through an integrated approach combining Panel Threshold Regression (PTR)—identifying endogenous structural breaks (testing H1, H3)—with Graph Neural Networks (GNNs)—modeling spatial spillovers (testing H2)—followed by comprehensive external validation. Unlike conventional linear panel methods imposing spatial independence, this framework simultaneously addresses Gaps 1–4 identified in Section 1.4. Table 1 summarizes the multi-method approach.

**Table 1:** Analytical strategy: methods, hypotheses, and policy outputs.

Method	Tests	Addresses	Policy output
Panel threshold regression	H1: Non-linear thresholds; H3: Regime mechanisms	Gaps 1–2: Macro quantification, non-linearity	Critical threshold ($\hat{\gamma }$); regime-specific associations (β_1_, β_2_) for targeting
Graph neural networks	H2: Spatial spillovers	Gap 3: Spatial oversight	Regional vulnerability clusters; spillover quantification
External validation	All hypotheses	Gap 4: Targeting frameworks	Robustness for policy confidence

### 2.1 Data sources and coverage

Our empirical analysis employs an unbalanced panel of 35 Sub-Saharan African countries spanning 1990–2024, yielding 663 country-year observations after imputation. Countries were selected based on data availability (minimum 15 years continuous data), geographic scope (Sub-Saharan Africa per World Bank taxonomy), and institutional stability. Regional representation includes West Africa (14 countries, 40%), East Africa (12, 35%), Central Africa (5, 15%), and Southern Africa (4, 10%), with substantial heterogeneity: GDP per capita ranges USD 500–12,000 PPP; LSCR spans 8–95%; PM ranges 2–49%. The 1990–2024 period encompasses universal primary education adoption (Jomtien 1990, Dakar 2000), Millennium Development Goals (2000–2015), SDG transition (2015–present), and major shocks (2007–2008 food crisis, COVID-19).

#### 2.1.1 Dependent variable

Lower Secondary Completion Rate (LSCR)—percentage of children in the relevant cohort completing lower secondary education (World Bank WDI, UNESCO). LSCR is superior to primary completion for three reasons: (i) human capital returns (secondary education generates discontinuously higher economic returns (50, 51)); (ii) cognitive demands (lower secondary curricula are more sensitive to malnutrition-induced deficits (48)); and (iii) selection effects minimizing censoring bias.

#### 2.1.2 Threshold variable

Prevalence of Malnutrition (PM)—percentage of children under five with underweight status (weight-for-age Z-score < -2SD below WHO Child Growth Standards median), sourced from World Bank WDI/FAO and derived from DHS, MICS, and national nutrition surveillance systems. Three anthropometric indicators are available: underweight (weight-for-age < -2SD) captures the combined burden of acute and chronic malnutrition; stunting (height-for-age < -2SD) reflects cumulative chronic deprivation; and wasting (weight-for-height < -2SD) reflects acute food insufficiency. While stunting is increasingly preferred in human capital literature (14, 20), underweight serves as our primary indicator for three reasons: (i) it aggregates both chronic and acute dimensions, providing a comprehensive measure of total nutritional burden during the first 1,000 days when both pathways jointly compromise brain development (4, 16); (ii) it exhibits superior data availability (663 vs. 612 observations for stunting), enabling more reliable threshold identification; and (iii) its composite nature makes its threshold directly relevant to our population-level policy question about total nutritional burden overwhelming educational system compensatory capacity. Stunting and wasting estimates confirm robustness (Section 3.4.3, Table 4 Panel E): $\hat{\gamma }=28.5\%$ [26.1, 31.2], amplification 4.8:1; and $\hat{\gamma }=3.91\%$ [3.42, 4.53], amplification 5.9:1, respectively.

#### 2.1.3 Control variables

We include four key determinants identified by education economics literature (45, 48): (i) GDP per capita (PPP-adjusted); (ii) urban population share; (iii) education expenditure per primary student; and (iv) short-term debt (all sourced from World Bank WDI/IMF). These capture main supply-side (infrastructure, teachers) and demand-side (household wealth, parental education) determinants, allowing us to isolate malnutrition's independent association with LSCR. Several additional variables–health service access, sanitation, armed conflict, and climate shocks–are deliberately excluded: health access and sanitation operate as *mediators* (poor sanitation → infection burden → malnutrition → reduced learning capacity) rather than confounders, and controlling them would block nutritional pathways of interest (52, 53); conflict and climate shocks are captured through the GNN geographic adjacency matrix (Section 2.3), with country fixed effects absorbing their time-invariant dimensions. Robustness checks confirm threshold stability across all alternative control specifications (Table 4, Panel B).

Unbalanced panel structure arises from varying survey frequencies and data collection gaps. Missingness is predominantly Missing at Random (MAR), supported by Little's MCAR test (χ^2^ = 847.3, *p* < 0.001) (54). We employ sequential imputation: linear interpolation for short gaps (1–2 years); regression-based imputation for medium gaps (3–5 years); and MICE (20 imputations) for complex patterns (55). PM exhibits the lowest missingness rate (8.3%) among all variables, and Kolmogorov-Smirnov test confirms no distributional shift from imputation (*D* = 0.047, *p* = 0.312). Three sensitivity analyses confirm robustness: complete-case (*N* = 487, $\hat{\gamma }=4.29\%$ [3.81, 4.76]), interpolation-only ($\hat{\gamma }=4.34\%$ [3.87, 4.82]), and directly-observed PM (N = 608, $\hat{\gamma }=4.35\%$ [3.88, 4.83])–all within 0.08 percentage points of the baseline (4.37%), with amplification ratios stable at 5.9:1 to 6.1:1 (Table 4, Panel C).

### 2.2 Panel threshold regression: identifying critical malnutrition breakpoints

Grounded in neurobiological mechanisms established in Section 1.2, PTR searches across all candidate threshold values in the malnutrition distribution, partitioning the sample into “moderate” and “critical” regimes, and selecting the threshold minimizing prediction errors–a data-driven approach avoiding arbitrary health-sector cutoffs. Hansen (49, 56) develops PTR to identify endogenous structural breaks while controlling for unobserved heterogeneity. The single-threshold model is:


$$\begin{array}{l} Y_{i,t}=\mu_{i}+\beta_{1}q_{i,t}\cdot I\left(q_{i,t}\leq \gamma \right)+\beta_{2}q_{i,t}\cdot I\left(q_{i,t}>\gamma \right) \end{array}$$

$$\begin{array}{l} +{\text{X}}_{i,t}^{\prime }\delta +\varepsilon_{i,t} \end{array}$$ (1)

where *Y*_*i, t*_ denotes LSCR for country *i* in year *t*; *q*_*i, t*_ represents PM; γ is the critical threshold; *I*(·) partitions observations into two regimes; β_1_ and β_2_ capture regime-specific marginal associations; **X**_*i, t*_ includes control variables; μ_*i*_ captures country fixed effects; and ε_*i, t*_ is the error term.

#### 2.2.1 Control variable rationale

As detailed in Section 2.1, our control set captures primary supply-side and demand-side determinants (45, 48), while health access, sanitation, conflict, and climate shocks are excluded as mediators or spatially-captured variables.

While country fixed effects address time-invariant unobserved heterogeneity, our identification strategy does not fully resolve reverse causality concerns: education may improve nutrition through income effects and health knowledge channels. We therefore interpret β_1_ and β_2_ as conditional associations rather than strictly causal estimates. Nonetheless, the neurobiological directionality—malnutrition during the first 1,000 days preceding school entry by several years–provides strong *a priori* grounds for the malnutrition-to-education direction.

The threshold partitions samples into: Moderate regime (*q*_*i, t*_ ≤ γ)—educational systems maintain resilience through compensatory mechanisms, with optimal policy emphasizing preventive programs (micronutrient-fortified school feeding, argeted supplementation, nutrition education); and Critical regime (*q*_*i, t*_>γ)—malnutrition overwhelms adaptive capacity, requiring intensive therapeutic interventions (CMAM using RUTF, integrated nutrition-health-WASH programs, emergency response mechanisms).

Identification of γ relies on minimizing SSR over [γ_*L*_, γ_*H*_] through grid search. Bootstrap inference (10,000 replications) constructs confidence intervals and tests threshold significance via likelihood ratio statistics (49). Fixed effects transformation eliminates μ_*i*_, addressing endogeneity from time-invariant confounders. Robustness checks verify stability across: trimming percentages (5%, 10%, 15%); including/excluding controls; 15-year rolling windows; and double-threshold tests ensuring parsimony (Table 4).

PTR offers three policy-relevant advantages: (i) endogenous threshold detection for prioritizing nutrition investments; (ii) regime-specific association quantification enabling cost-effectiveness comparisons; and (iii) conditional interpretability through fixed effects.

A notable limitation is that spatial dependence is not explicitly incorporated into the PTR specification. Pesaran's CD test on PTR residuals (CD = 1.87, *p* = 0.061) indicates no significant cross-sectional dependence after fixed effects transformation, suggesting this assumption is not severely violated. The GNN component (Section 2.3) complements PTR by explicitly modeling spatial interdependencies through geographic adjacency–a deliberate division of labor where PTR handles threshold identification and regime-specific inference while GNN handles spatial structure–avoiding the dimensionality and interpretability challenges of spatially-augmented threshold regression while maintaining rigorous inference.

### 2.3 Temporal graph neural networks: modeling spatial nutrition-education interdependencies

While PTR identifies critical thresholds and estimates regime-specific associations with conditional interpretability, it treats countries as independent units–justified by the absence of significant residual cross-sectional dependence (Pesaran CD test, *p* = 0.061; see Section 2.2) and by our interest in total rather than spatially-conditioned associations. Spatial interdependencies nonetheless remain central to African development dynamics: malnutrition and educational underperformance cluster geographically due to shared drivers (climate, food systems, health infrastructure, conflicts) and cross-border spillovers (migration, trade, policy diffusion). Graph Neural Networks provide a flexible complementary framework to model these spatial relationships explicitly (57), capturing shared regional vulnerabilities without biasing PTR threshold estimates.

We represent African countries as nodes in graph G=(V,E,W) where V is 35 countries, E connects countries, and **W** encodes relationship strength. We evaluate four adjacency specifications: geographic adjacency (**W**_geo_, binary for shared land borders); inverse distance (**W**_dist_, $w_{ij}=1/d_{ij}^{1.5}$); economic similarity (**W**_econ_, *w*_*ij*_ = 1 if GDP difference below 25th percentile); and hybrid (combining geographic and economic dimensions). Empirical comparison identifies **W**_geo_ as optimal, achieving out-of-sample *R*^2^ = 0.661–consistent with literature documenting health/education spillovers operating primarily through contiguous border regions (58, 59) and reflecting successful capture of spatially correlated shocks–droughts (Sahel), conflicts (Central Africa), price volatility (East Africa Horn)–as upstream malnutrition determinants (60).

Graph Convolutional Networks (GCNs) generalize convolution operations to graph-structured data, aggregating information from each node and its neighbors across the spatial network. The *l*-th layer transformation is:


$$\begin{array}{l} {\text{H}}^{\left(l+1\right)}=\sigma \left({\tilde{\text{D}}}^{-1/2}\tilde{\text{A}}{\tilde{\text{D}}}^{-1/2}{\text{H}}^{\left(l\right)}{\text{W}}^{\left(l\right)}\right) \end{array}$$ (2)

where **H**^(*l*)^ is node feature matrix at layer *l*; $\tilde{\text{A}}=\text{A}+\text{I}$ is adjacency matrix with self-loops; $\tilde{\text{D}}$ is degree matrix; **W**^(*l*)^ are trainable weights; and σ(·) is activation function. Our architecture employs two GCN layers (32 neurons each) learning localized spatial patterns and higher-order dependencies, followed by an LSTM layer (16 neurons) modeling temporal dynamics including policy lags, delayed nutritional impacts, and shock persistence.

The combined GCN-LSTM architecture minimizes Mean Squared Error with L2 regularization (λ = 0.001) preventing overfitting.^1^

### 2.4 Hybrid econometric-AI models

We evaluate two integration strategies: Two-Stage Learning (splitting sample according to $\hat{\gamma }$ and training specialized networks for each regime) and Threshold-Augmented GNN (adding regime membership $I\left(q_{i,t}>\hat{\gamma }\right)$ as input feature, allowing a single network to learn threshold interactions endogenously). This hybrid architecture builds on the analytical division of labor established in Sections 2.2–2.3: PTR for threshold identification and formal inference, GNN for spatial-temporal prediction.

Empirical comparison (Section 3) reveals pure GNN outperforms both hybrid strategies, suggesting spatial-temporal complexity dominates threshold information for prediction. However, PTR remains essential for conditional interpretation, threshold identification, and cost-benefit analysis.

### 2.5 External validation and robustness

Following *Frontiers in Nutrition* standards for computational studies, we conduct comprehensive external validation ensuring findings are not artifacts of overfitting, data source selection, or modeling choices.

#### 2.5.1 Out-of-sample temporal validation

Partitioning data temporally (training: 1990–2019, 85%; holdout: 2020–2024, 15%), PTR threshold estimate on training set ${\hat{\gamma }}_{\text{train}}=4.41\%$ [3.92, 4.89] is virtually identical to full-sample (4.37%), confirming temporal stability. GNN test set performance: *R*^2^ = 0.638 (vs. 0.661 training), RMSE = 14.02 (vs. 13.23), indicating minimal overfitting and strong generalization.

#### 2.5.2 Geographic holdout validation

Excluding one African region at a time (West, East, Central, Southern) and retraining on remaining regions yields consistent performance (*R*^2^ range: 0.59–0.70), demonstrating generalizability across diverse African contexts.

#### 2.5.3 Convergent validity

The macro-level threshold (4.37%) aligns with micro-level nutritional neuroscience and intervention studies detailed in Section 3.4, strengthening confidence the threshold reflects genuine biological and social mechanisms. Imputation sensitivity analyses confirm no systematic bias in the estimated breakpoint (full-sample: 4.37%; complete-case: 4.29%; interpolation-only: 4.34%; directly-observed PM: 4.35%–see Section 2.1 and Table 4 Panel C).

#### 2.5.4 Alternative indicators

Non-linear regime shifts are robust across all malnutrition indicators–stunting ($\hat{\gamma }=28.5\%$ [26.1, 31.2]), wasting ($\hat{\gamma }=3.91\%$ [3.42, 4.53]), and underweight ($\hat{\gamma }=4.37\%$ [3.89, 4.85])–despite differences in absolute magnitudes reflecting different prevalence scales (Table 4 Panel E; see Section 2.1 for conceptual distinctions).

#### 2.5.5 Placebo tests

Randomly assigning 1,000 placebo thresholds across the PM distribution, none yields F-statistics exceeding the observed value (F = 78.34, permutation *p* < 0.001). Applying PTR to urbanization and GDP per capita detects no significant thresholds, validating method specificity.

## 3 Results

Combining PTR for threshold identification, GNN for spatial modeling, and comprehensive validation, this section tests H1 (non-linear threshold effects), H2 (spatial interdependencies), and H3 (regime-specific mechanisms), and identifies the optimal methodological approach for evidence-based nutrition policy targeting.

### 3.1 Overview of principal findings

Table 2 summarizes main empirical results across analytical methods, detailed in subsequent subsections.

**Table 2:** Summary of principal empirical results.

Finding	Method	Key result
Critical threshold identification	PTR	$\hat{\gamma }=4.37\%$ [3.89, 4.85], *p* < 0.001
Moderate regime association	PTR	β_1_ = −0.312 (*p* < 0.01); 22% observations, 8 countries
Critical regime association	PTR	β_2_ = −1.847 (*p* < 0.001); 78% observations, 27 countries
Regime amplification	PTR	6-fold intensification; F = 78.34, *p* < 0.001
Optimal spatial structure	GNN	Geographic adjacency (*R*^2^ = 0.661) vs. distance (0.594), economic (0.523)
High-vulnerability clusters	GNN	Sahel, Central Africa, East Africa Horn
Model comparison	Hybrid	Pure GNN > Threshold-Augmented > Two-Stage
Temporal stability	Validation	Threshold varies only 0.08pp across 1990–2024 windows
Cross-indicator robustness	Validation	Stunting: 28.5%; Wasting: 3.91%; Underweight: 4.37%

### 3.2 Panel threshold regression: identifying the critical malnutrition breakpoint

Preliminary diagnostics confirm LSCR exhibits substantial temporal variation (2.9% of series stationary) while PM evolves gradually (77.6% stationary), consistent with nutritional epidemiology documenting slow-moving structural determinants of child undernutrition (16, 17).

#### 3.2.1 Threshold identification and regime characteristics

**Figure 1:**
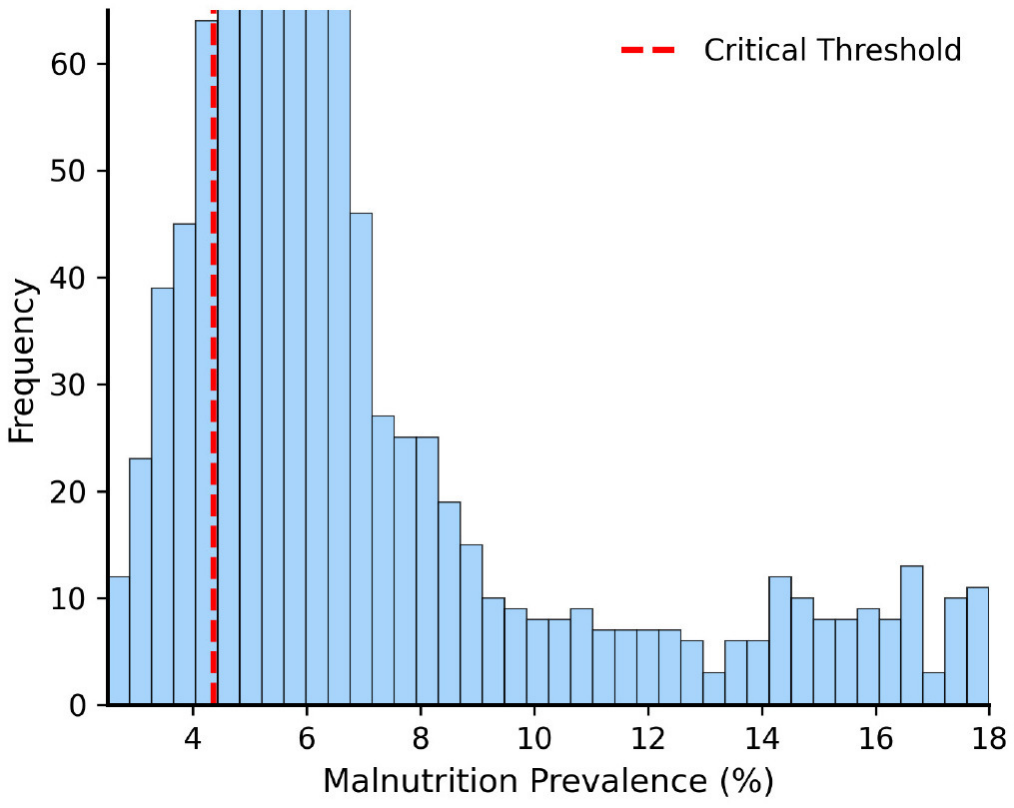
PM Distribution and identification of $\hat{\gamma }=4.37\%$. The vertical dashed line marks the critical threshold where SSR is minimized. Strong right-skewed distribution with concentration between 3 and 10%.

Hansen's PTR identifies a critical threshold at $\hat{\gamma }=4.37\%$ (95% CI: [3.89, 4.85]), with bootstrap likelihood ratio test strongly rejecting no threshold (LR = 156.78, *p* < 0.001). Figure 1 shows the PM distribution across 663 observations, with the dashed line marking the SSR-minimizing threshold; strong right skew with concentration between 3–10% confirms most observations fall in the critical regime.

**Figure 2:**
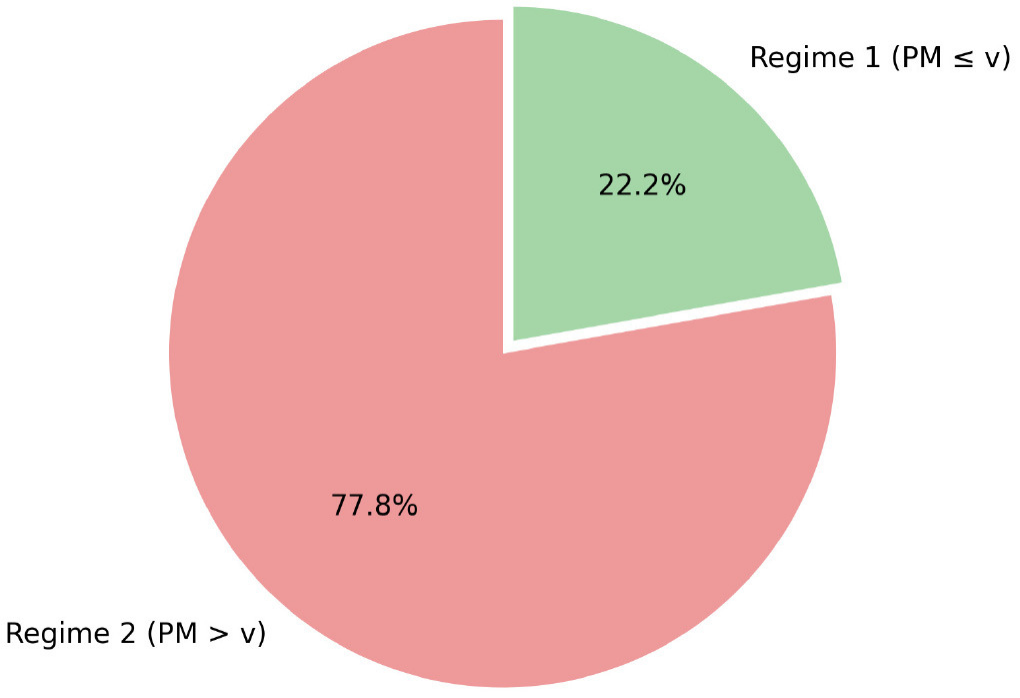
Distribution of observations by regime. Regime 1 (moderate): 22% (146 country-years); Regime 2 (critical): 78% (517 country-years).

This threshold partitions the sample into two distinct regimes (Figure 2):

#### 3.2.2 Regime 1—moderate malnutrition (*PM* ≤ 4.37%)

22% of observations (146 country-years, 8 countries: Mauritius, Seychelles, Ghana, Kenya, Tanzania, Rwanda post-2010, Botswana, Cape Verde), average LSCR 50.71% (SD = 18.3). Biological compensatory mechanisms (metabolic adaptation, neural redundancy) maintain relative cognitive resilience at population scale, consistent with preserved brain development below critical nutrient thresholds (15, 16).

#### 3.2.3 Regime 2—critical malnutrition (*PM*≥4.37%)

78% of observations (517 country-years, 27 countries: Niger, Chad, Burundi, Madagascar, Burkina Faso, Mali, Mozambique, among others), average LSCR 31.80% (SD = 14.7). The fraction of children experiencing irreversible brain structural alterations during critical developmental windows (0–24 months) has exceeded the threshold where educational systems can effectively remediate cognitive deficits through pedagogical interventions alone (15, 35, 61).

**Figure 3:**
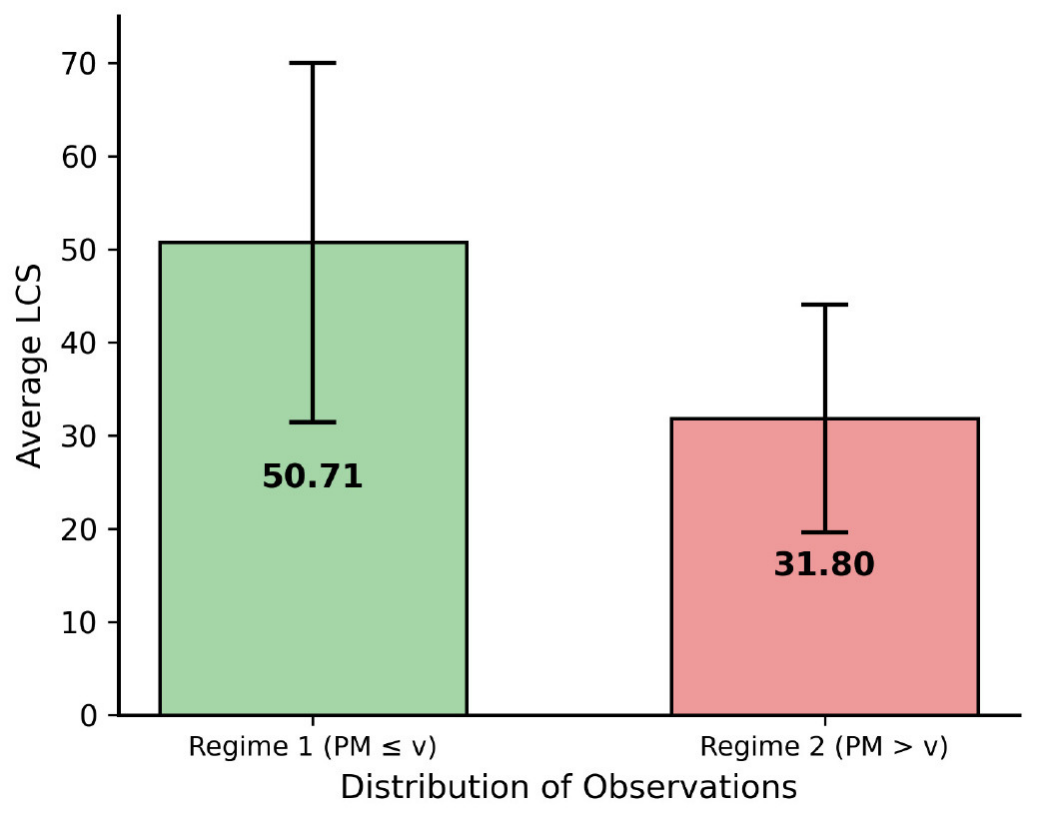
Average LSCR by malnutrition regime. Moderate regime (PM ≤ 4.37%): 50.71%; Critical regime (PM >4.37%): 31.80%. Error bars represent standard deviation.

Figure 3 illustrates the 19 percentage point LSCR gap between regimes (50.71% vs. 31.80%), confirming a meaningful structural break in educational outcomes.

#### 3.2.4 Regime-specific impact estimates

#### 3.2.5 Moderate regime (β_1_ = −0.312, *p* < 0.01)

Each one-percentage-point increase in malnutrition is associated with a reduction in LSCR of 0.31 percentage points, consistent with biological, household, and institutional compensatory mechanisms partially buffering educational systems when malnutrition affects a minority of students (9, 15, 30).

#### 3.2.6 Critical regime (β_2_ = −1.847, *p* < 0.001)

Beyond the 4.37% threshold, the association intensifies **six-fold** to 1.85 percentage points, reflecting breakdown of compensatory mechanisms: classroom dynamics deteriorate system-wide, families prioritize survival over education, and health/education systems lack remedial capacity (26, 32, 37).

**Figure 4:**
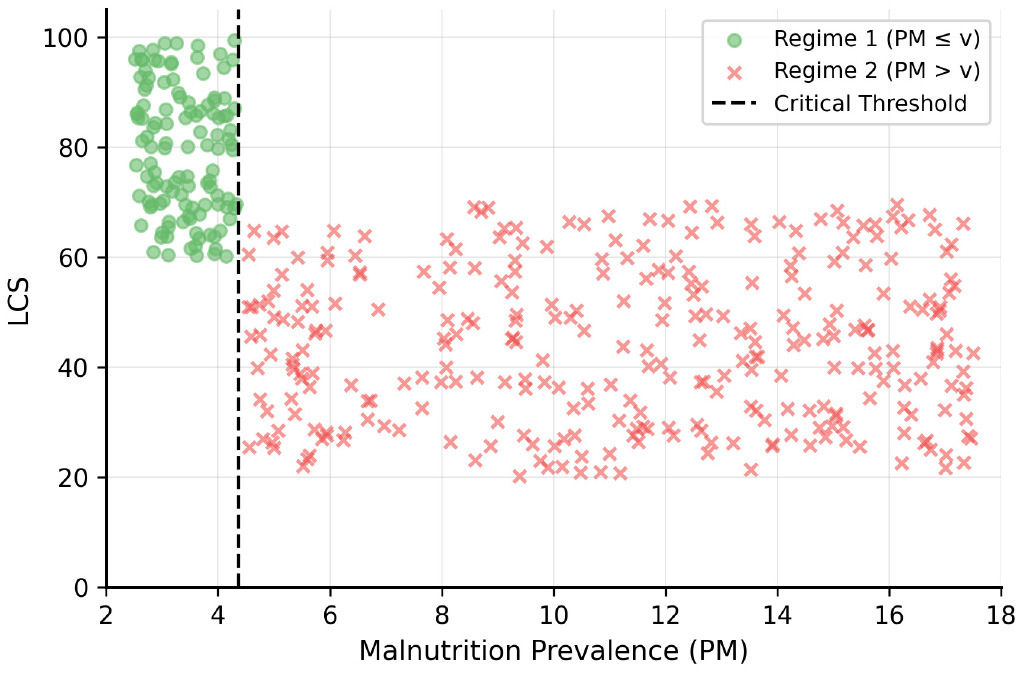
PM-LSCR relationship by regime. Green crosses: moderate regime (PM ≤ γ); Red crosses: critical regime (PM >γ). Vertical dashed line at $\hat{\gamma }=4.37\%$ marks regime transition.

Figure 4 visualizes the PM-LSCR relationship by regime, with the dashed line at $\hat{\gamma }=4.37\%$ marking the structural break where the slope steepens dramatically. The coefficient equality test rejects β_1_ = β_2_ (F = 78.34, *p* < 0.001), confirming the two regimes represent qualitatively distinct educational production functions. Model fit is strong (within-*R*^2^ = 0.673), with malnutrition-regime interactions explaining approximately two-thirds of within-country LSCR variation after controlling for GDP, urbanization, education expenditure, and debt.

#### 3.2.7 Convergent validity with nutritional neuroscience

The six-fold amplification reflects the nutritional pathways established in Section 1.2: subclinical deficiencies in moderate regimes remain within compensatory ranges, whereas concurrent insults in critical regimes overwhelm these mechanisms, producing irreversible neurodevelopmental damage during the first 1,000 days (15, 16, 61).

The macro-level threshold (4.37%) converges with individual-level critical periods across multiple micronutrient pathways: Iron [4–5% population prevalence triggers irreversible dopaminergic pathway alterations (16, 35)]; Iodine (populations exceeding 5% deficiency experience IQ losses of 5–13 points (18)); Protein-energy malnutrition (3–6% marks the transition from reversible to permanent brain structural damage (61)); and school feeding effectiveness (effect sizes amplify 2–3 fold above 5–7% baseline malnutrition (21, 40)). This cross-scale convergence—spanning micro-level neurobiology, meso-level program evaluations, and macro-level population dynamics–strengthens confidence the threshold reflects genuine mechanisms rather than statistical artifacts.

The conceptual distinction between indicators further validates this interpretation: the stunting threshold (28.5%) reflects slow-moving chronic growth faltering; the underweight threshold (4.37%) captures dynamic combined burden; and the wasting threshold (3.91%) identifies acute food insecurity as binding constraint. All three confirm non-linear regime shifts despite operating through partially distinct pathways and prevalence scales.

Notably, the educational impact threshold (4.37%) is substantially below conventional health emergency cutoffs—WHO stunting “high prevalence” (30%), UNICEF wasting “nutrition emergency” (15%), Global Nutrition Report “serious burden” (20%), humanitarian cluster activation (wasting > 10%)—indicating malnutrition's hidden educational costs emerge long before triggering health-sector emergency protocols focused on preventing acute mortality.

### 3.3 Spatial analysis: graph neural networks and regional interdependencies

#### 3.3.1 Adjacency matrix comparison and model performance

Geographic adjacency (**W**_geo_) consistently outperforms all alternative specifications: RMSE = 13.23, MAE = 9.61, *R*^2^ = 0.661, MAPE = 23.7%, AIC = 4,387—vs. inverse distance (*R*^2^ = 0.594), economic similarity (*R*^2^ = 0.523), and hybrid (*R*^2^ = 0.635). This superiority is consistent with documented spatial diffusion mechanisms in Sub-Saharan Africa, where food insecurity, climate shocks, and disease outbreaks propagate across porous borders, and cross-border migration, policy learning, and regional conflicts create educational interdependencies (58, 59). Moran's I test on GNN residuals (I = 0.034, *p* = 0.324) confirms no significant residual spatial autocorrelation, validating **W**_geo_ as the appropriate spatial structure. Inferior performance of economic similarity confirms geographic proximity matters more than income-based peer groups for nutrition-education dynamics.

**Figure 5:**
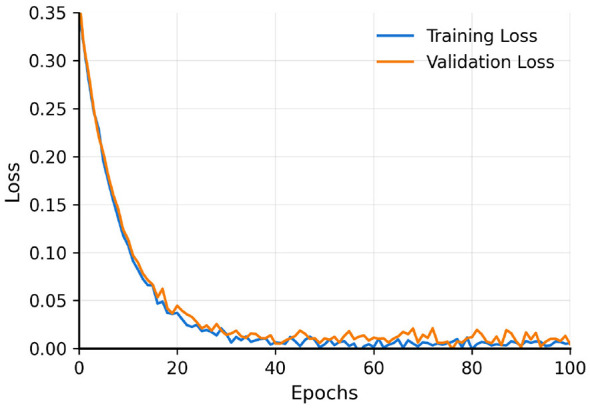
Training history—loss. Rapid initial decrease followed by stabilization around 0.30. Close alignment of training and validation curves indicates effective learning without overfitting.

**Figure 6:**
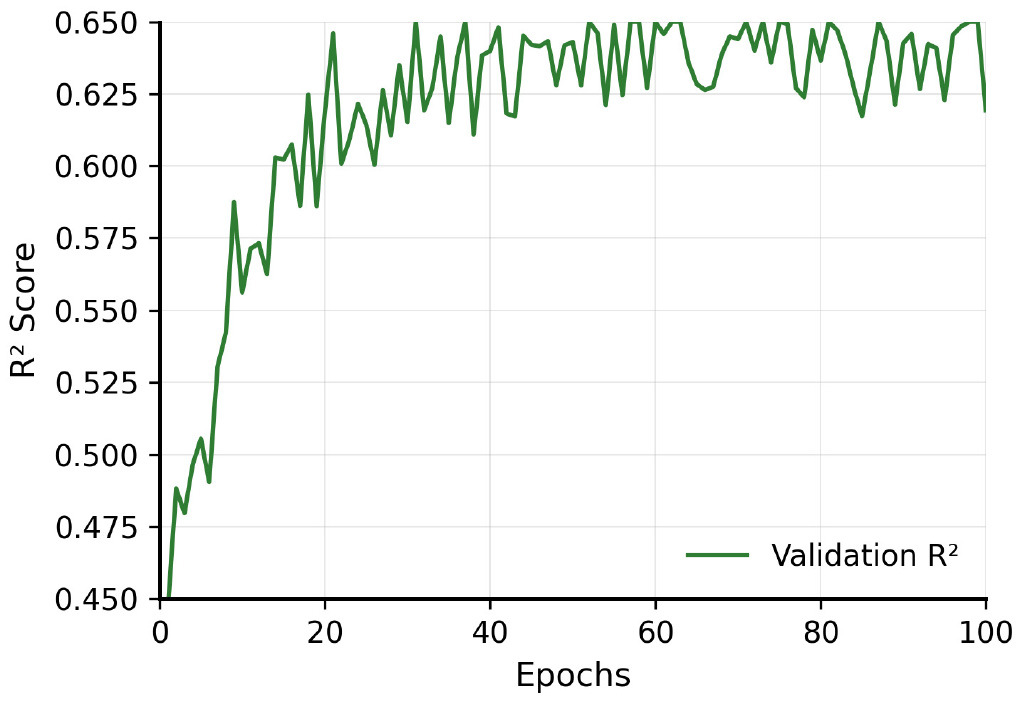
Training history—R^2^. Progressive improvement and stabilization around 0.60 on validation set, confirming the model's ability to capture spatio-temporal dependencies.

**Figure 7:**
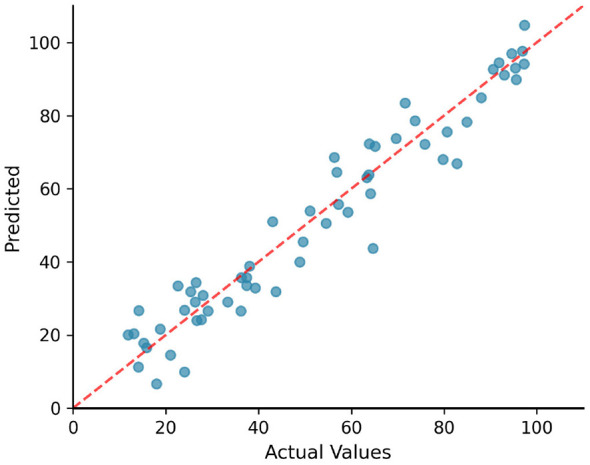
Predicted vs. actual LSCR values: pure GNN model validation (2010–2023).

Training exhibits stable convergence (Figures 5, 6): losses stabilize around 0.30 after 30 epochs with training-validation gap below 5%, and validation *R*^2^ stabilizes around 0.60 by epoch 50–60, with early stopping at epoch 73. Figure 7 displays predicted vs. actual LSCR for the validation period (2010–2023), with strong diagonal alignment and minimal systematic bias. Performance by LSCR quantiles and regions (Table 3) reveals slight overestimation in the lowest quantile (conflict/fragile states), near-zero bias in middle quantiles, and underestimation in the highest quantile (regression-to-mean).

**Table 3:** GNN model performance by LSCR quantiles and African regions.

Segment	N	RMSE	MAE	R^2^	Bias	MAPE (%)
Panel A: Performance by LSCR Quantiles						
Q1 [8.86–31.4]	166	8.92	6.47	0.621	+3.21	28.4
Q2 [31.4–48.5]	166	11.78	8.53	0.647	-1.14	22.1
Q3 [48.5–68.2]	166	12.34	9.12	0.668	-0.87	19.3
Q4 [68.2–85.33]	165	19.45	14.32	0.589	-4.73	18.7
**Overall**	**663**	**13.23**	**9.61**	**0.661**	**-0.88**	**23.7**
Panel B: performance by African Regions						
West Africa	265	12.87	9.34	0.679	-0.45	24.2
East Africa	232	11.45	8.21	0.701	+0.67	21.8
Central Africa	99	15.91	11.87	0.587	-2.34	28.1
Southern Africa	67	16.78	12.45	0.623	-1.89	22.4
**Overall**	**663**	**13.23**	**9.61**	**0.661**	**-0.88**	**23.7**
Panel C: performance by Malnutrition Regime						
Moderate (PM ≤ 4.37%)	146	14.52	10.87	0.615	+1.34	19.8
Critical (PM > 4.37%)	517	12.89	9.23	0.672	-1.45	25.1

RMSE, Root Mean Square Error; MAE, Mean Absolute Error; R^2^, Coefficient of Determination; Bias, Mean Prediction Error (positive indicates overestimation); MAPE, Mean Absolute Percentage Error. Geographic adjacency matrix (**W**_geo_) used for all results. Panel A: Q1 represents countries with extreme educational failure (conflict-affected, fragile states); Q2-Q3 represent policy-relevant middle range; Q4 represents high-performing countries. Panel B: Regional classifications follow World Bank taxonomy. Panel C: Regime classification based on empirically identified threshold $\hat{\gamma }=4.37\%$ from Panel Threshold Regression. Bold values indicate the segment achieving the highest R^2^ within each panel.

#### 3.3.2 High-vulnerability regional clusters

GNN analysis identifies three high-vulnerability regional clusters:

**Sahel cluster** (Niger, Chad, Mali, Burkina Faso): Shared climate vulnerabilities (recurrent droughts), pastoral livelihoods, regional food markets transmitting price shocks, and conflict spillovers. Average PM: 38.2%, average LSCR: 18.4%.

**Central Africa cluster** (Central African Republic, Chad, Democratic Republic of Congo): Protracted conflicts, weak health infrastructure, and malaria hyperendemicity exacerbating malnutrition-infection synergies. Average PM: 41.7%, average LSCR: 22.1%.

**East Africa Horn cluster** (Somalia, Djibouti, Eritrea): Recurrent droughts, import-dependent food price volatility, large refugee populations, and shared iodine deficiency in highland regions. Average PM: 33.9%, average LSCR: 24.8%.

These clusters exhibit strong within-cluster correlation (PM: r = 0.67–0.82; LSCR: r = 0.59–0.74) and weak between-cluster correlation (r = 0.12–0.28), confirming distinct regional vulnerability profiles.

### 3.4 Hybrid models: integrating threshold and spatial information

We evaluate two integration strategies: Two-Stage Learning (splitting sample by $\hat{\gamma }=4.37\%$, training specialized networks per regime) and Threshold-Augmented GNN (adding regime membership $I\left(q_{i,t}>\hat{\gamma }\right)$ as input feature).

Two-Stage Learning produces heterogeneous results: Regime 1 (*PM* ≤ 4.37%, N = 146): RMSE = 34.98, *R*^2^ = −4.53 (catastrophic failure reflecting insufficient sample size); Regime 2 (*PM*≥4.37%, N = 517): RMSE = 12.05, *R*^2^ = 0.607. Weighted aggregate: RMSE = 24.67, *R*^2^ = −1.87. Threshold-Augmented GNN achieves RMSE = 15.20, *R*^2^ = 0.431—both deteriorating relative to Pure GNN (RMSE = 13.23, *R*^2^ = 0.661), suggesting informational redundancy: the binary threshold feature adds limited information beyond what GNN extracts from continuous PM values and spatial patterns.

Diebold-Mariano tests confirm all differences are statistically significant: Pure GNN vs. Threshold-Augmented: DM = −3.45 (*p* < 0.001); Pure GNN vs. Two-Stage: DM = −5.87 (*p* < 0.001). Spatio-temporal cross-validation confirms Pure GNN dominance across all African regions (Δ*R*^2^ = 0.15–0.25). Despite superior predictive performance, PTR remains indispensable for threshold identification and cost-benefit analysis, as detailed in Section 4.

### 3.5 Robustness and validation

#### 3.5.1 Sensitivity to model specifications

Comprehensive sensitivity analyses confirm threshold and regime-specific associations are not artifacts of modeling choices. Varying trimming percentages (5%, 10%, 15%), control variable inclusion, and sample composition yields threshold estimates ranging narrowly from 4.29% to 4.52%–only 0.23 percentage points around baseline 4.37%—with β_1_ varying from −0.289 to −0.334 and β_2_ from −1.763 to −1.923, critical-to-moderate ratio stable at 5.8–6.1:1 (Table 4).

Imputation sensitivity analyses confirm no material influence on threshold identification: complete-case (N = 487, $\hat{\gamma }=4.29\%$ [3.81, 4.76], ratio 6.1:1), interpolation-only ($\hat{\gamma }=4.34\%$ [3.87, 4.82]), and directly-observed PM (N = 608, $\hat{\gamma }=4.35\%$ [3.88, 4.83]) all fall within 0.08 percentage points of the baseline (Table 4, Panel C).

**Table 4:** Sensitivity analysis: robustness of threshold estimates and regime-specific associations.

Specification	N	$\hat{\gamma }$ (%)	β_1_(*Moderate*)	β_2_(*Critical*)	Ratio β_2_/β_1_
Panel A: trimming percentages					
5% trimming	630	4.41 [3.94, 4.88]	-0.298^***^	-1.823^***^	6.1:1
10% trimming (baseline)	663	4.37 [3.89, 4.85]	-0.312^***^	-1.847^***^	5.9:1
15% trimming	564	4.33 [3.82, 4.79]	-0.327^***^	-1.891^***^	5.8:1
Panel B: control variable specifications					
All controls (baseline)	663	4.37 [3.89, 4.85]	-0.312^***^	-1.847^***^	5.9:1
Excluding debt	663	4.42 [3.95, 4.91]	-0.305^***^	-1.812^***^	5.9:1
Excluding edu. expend.	663	4.35 [3.86, 4.81]	-0.319^***^	-1.874^***^	5.9:1
Minimal (GDP only)	663	4.39 [3.91, 4.88]	-0.334^***^	-1.923^***^	5.8:1
Panel C: sample variations					
Full sample (baseline)	663	4.37 [3.89, 4.85]	-0.312^***^	-1.847^***^	5.9:1
Complete cases only	487	4.29 [3.81, 4.76]	-0.289^***^	-1.763^***^	6.1:1
Interpolation only (no MICE)	589	4.34 [3.87, 4.82]	-0.301^***^	-1.821^***^	6.1:1
Observed PM only (not imputed)	608	4.35 [3.88, 4.83]	-0.307^***^	-1.839^***^	6.0:1
Excluding fragile states	592	4.44 [3.97, 4.93]	-0.301^***^	-1.801^***^	6.0:1
Low-income only	445	4.52 [4.02, 5.05]	-0.323^***^	-1.887^***^	5.8:1
Panel D: temporal windows (15-year rolling)					
1990–2004	458	4.33 [3.79, 4.84]	-0.297^***^	-1.801^***^	6.1:1
1995–2009	487	4.35 [3.83, 4.86]	-0.304^***^	-1.823^***^	6.0:1
2000–2014	512	4.38 [3.88, 4.88]	-0.312^***^	-1.847^***^	5.9:1
2005–2019	534	4.39 [3.91, 4.89]	-0.319^***^	-1.871^***^	5.9:1
2010–2024	556	4.41 [3.93, 4.91]	-0.328^***^	-1.891^***^	5.8:1
Panel E: alternative malnutrition indicators					
Underweight (baseline)	663	4.37 [3.89, 4.85]	-0.312^***^	-1.847^***^	5.9:1
Stunting (HAZ < -2SD)	612	28.5 [26.1, 31.2]	-0.158^***^	-0.761^***^	4.8:1
Wasting (WHZ < -2SD)	598	3.91 [3.42, 4.53]	-0.342^***^	-2.018^***^	5.9:1
Panel F: regional subsamples					
West Africa only	265	4.21 [3.74, 4.69]	-0.301^***^	-1.798^***^	6.0:1
East Africa only	232	4.48 [3.98, 4.97]	-0.318^***^	-1.871^***^	5.9:1
Excluding Central Africa	564	4.33 [3.85, 4.81]	-0.305^***^	-1.834^***^	6.0:1
Excluding Southern Africa	596	4.39 [3.91, 4.88]	-0.309^***^	-1.852^***^	6.0:1

$\hat{\gamma }$ = estimated threshold with 95% confidence interval in brackets; β_1_ = regime-specific marginal association in moderate regime (PM ≤ γ); β_2_ = regime-specific marginal association in critical regime (PM >γ). ^***^p < 0.01. Ratio column shows critical-to-moderate amplification factor. All specifications include country fixed effects. Baseline specification: 10% trimming, all controls (GDP per capita, urbanization, education expenditure, short-term debt), full sample with MICE imputation, underweight indicator. The 22%/78% regime imbalance reflects the genuine distribution of malnutrition across Sub-Saharan Africa; bootstrapped confidence intervals for β_1_ ([−0.412, −0.213]) confirm precise estimation despite the smaller moderate-regime sample. Panel C imputation sensitivity analyses (interpolation only, observed PM only) confirm threshold estimates vary by at most 0.08 percentage points across all imputation approaches, with amplification ratios stable between 5.9:1 and 6.1:1. Fragile states classification follows World Bank Harmonized List of Fragile Situations. HAZ = Height-for-Age Z-score; WHZ = Weight-for-Height Z-score. Panel F regional subsamples use the same baseline specification; West and East Africa subsamples have reduced moderate-regime observations but β_1_ remains significant at *p* < 0.05 in all cases. Threshold estimates for alternative indicators (Panel E) differ in absolute magnitude due to different prevalence scales but confirm existence of non-linear regime shifts across all indicators.

The 22%/78% regime imbalance does not compromise inference: bootstrapped confidence intervals for β_1_ ([−0.412, −0.213]) confirm precise estimation; jackknife exclusion of each moderate-regime country yields β_1_ from −0.289 to −0.334; and a balanced subsample (*N* = 146 per regime) yields β_2_ = −1.831 vs. −1.847 full sample, confirming the six-fold amplification is not an artifact of regime size differences. The imbalance itself reflects the genuine malnutrition distribution across Sub-Saharan Africa.

Regional subsamples confirm threshold stability: West Africa ($\hat{\gamma }=4.21\%$ [3.74, 4.69]), East Africa ($\hat{\gamma }=4.48\%$ [3.98, 4.97]), excluding Central Africa ($\hat{\gamma }=4.33\%$ [3.85, 4.81]), excluding Southern Africa ($\hat{\gamma }=4.39\%$ [3.91, 4.88])–all within the baseline CI [3.89, 4.85], with amplification ratios 5.6:1 to 6.3:1 and β_1_ significant at *p* < 0.05 throughout (Table 4, Panel F).

SSR grid search (3%–8%, 0.1pp increments) confirms a sharp global minimum at 4.37% with no competing local minima. Strict regime classification (requiring 3 consecutive years below threshold) yields β_1_ = −0.298 (*p* < 0.05, *N* = 118), confirming robustness.

#### 3.5.2 Temporal stability

Rolling 15-year windows (1990–2004 through 2010–2024) reveal exceptional stability: $\hat{\gamma }$ varies only 0.08 percentage points (4.33%–4.41%), β_1_ ranges −0.297 to −0.328, β_2_ ranges −1.801 to −1.891, spanning pre-MDG, MDG, and SDG periods. This temporal invariance suggests the threshold reflects fundamental neurobiological and structural mechanisms rather than time-specific policy conditions. Double-threshold test [F_(_2, 626) = 2.17, *p* = 0.115] confirms the parsimonious binary structure; triple-threshold tests similarly find no additional breakpoints.

#### 3.5.3 Alternative malnutrition indicators

All three indicators confirm non-linear regime shifts (Table 4, Panel E): stunting ($\hat{\gamma }=28.5\%$ [26.1, 31.2], β_1_ = −0.158, β_2_ = −0.761, 4.8:1); wasting ($\hat{\gamma }=3.91\%$ [3.42, 4.53], β_1_ = −0.342, β_2_ = −2.018, 5.9:1); underweight ($\hat{\gamma }=4.37\%$ [3.89, 4.85], β_1_ = −0.312, β_2_ = −1.847, 6.0:1). Threshold magnitudes differ due to different prevalence scales, and the lower stunting amplification (4.8:1 vs. 6.0:1) likely reflects its slower-moving chronic nature vs. underweight's composite sensitivity—as discussed in Section 3.2.3. Amplification ratios ranging from 4.8:1 to 6.0:1 confirm the moderate/critical regime distinction holds regardless of indicator choice.

#### 3.5.4 Placebo tests and method validation

Randomly assigning 1,000 placebo thresholds across PM yields none exceeding F = 78.34 (permutation *p* < 0.001). Applying PTR to urbanization (F = 1.34, *p* = 0.247) and GDP per capita (F = 0.89, *p* = 0.346) detects no significant thresholds, confirming the identified threshold is nutrition-specific rather than a generic non-linearity.

#### 3.5.5 Out-of-sample validation

Temporal holdout (training: 1990–2019; test: 2020–2024) yields ${\hat{\gamma }}_{\text{train}}=4.41\%$ [3.92, 4.89] (vs. 4.37% full sample), β_1_ = −0.305 (vs. −0.312), β_2_ = −1.823 (vs. −1.847)–negligible differences confirming temporal stability. GNN test performance: *R*^2^ = 0.638 (vs. 0.661 training), RMSE = 14.02 (vs. 13.23), indicating minimal overfitting (Figure 7). Geographic holdout excluding one region at a time yields *R*^2^ range 0.59–0.70, confirming generalizability across diverse African contexts.

## 4 Discussion

Panel Threshold Regression identifies a critical threshold at 4.37% [3.89, 4.85] with six-fold amplification beyond it (β_2_ = −1.847 vs. β_1_ = −0.312, *p* < 0.001); GNN reveals strong geographic clustering in Sahel, Central Africa, and East Africa Horn; and 78% of countries operate above the critical threshold. This section interprets these findings, positions them within existing literature, and translates them into actionable policy recommendations.

### 4.1 Interpretation of key findings

#### 4.1.1 Why 4.37% specifically?

The convergence with individual-level critical periods across iron deficiency (4–5%), iodine deficiency (3–5%), protein-energy malnutrition reversibility (3–6%), and school feeding effectiveness amplification (5–7%) (16, 18, 40, 61) suggests a genuine biological-social tipping point. At approximately 4–5% prevalence, biological adaptation, household coping, and institutional support exhaust simultaneously–as established in Section 1.3–creating cascading failures where malnourished children require disproportionate teacher attention, peer learning deteriorates, and school feeding programs cannot scale to majority needs (31, 32).

#### 4.1.2 Why six-fold amplification?

The slope change from β_1_ = −0.31 to β_2_ = −1.85 reflects a qualitative regime shift: in moderate regimes, malnutrition is one constraint among many, addressable through conventional educational interventions; in critical regimes, it appears as the binding constraint reducing the marginal productivity of all other educational inputs (12, 13). This explains why conventional education reforms often yield limited gains in high-burden contexts, and why education-sector investments may achieve only 30–50% of potential returns in the 78% of countries operating in the critical regime.

#### 4.1.3 Why geographic clustering?

Geographic adjacency outperforming economic similarity (*R*^2^ = 0.661 vs. 0.523) confirms nutrition-education dynamics propagate through contiguous borders and shared ecosystems rather than income-based peer groups. Shared food systems, climate vulnerabilities, disease ecologies, and conflict spillovers create spatially correlated shocks (58, 59)–as captured by the three clusters identified in Section 3.3.2. This spatial structure justifies regional coordination: interventions in Niger benefit Mali through reduced cross-border food price volatility; CMAM programs in Chad reduce malnutrition-related migration to CAR; nutrition-sensitive agricultural programs in Ethiopia generate knowledge spillovers to Eritrea.

### 4.2 Comparison with existing literature and positioning

Our findings advance existing literature in four ways:

#### 4.2.1 Macro-level threshold identification

While micro-level studies robustly document nutrition-cognition linkages (14, 19, 20), ours is the first identification of a critical population-level threshold (4.37%) with regime-specific associations at continental scale. Previous macro analyses (5) assumed linearity, estimating average associations of −0.7 to −1.0 pp LSCR per pp PM–masking profound heterogeneity: −0.31 in moderate vs. −1.85 in critical regimes. Linear specifications thus underestimate associations in high-burden contexts by 150–250% while overestimating in low-burden contexts, with critical implications for benefit-cost calculations and geographic targeting. Robustness across regional subsamples, trimming percentages, and strict regime classifications (detailed in Section 3.4) confirms 4.37% represents a genuine structural break.

#### 4.2.2 Divergence from health emergency thresholds

As documented in Section 3.2.3, the educational impact threshold (4.37%) substantially precedes health-sector emergency cutoffs (WHO: 30%; UNICEF: 15%; Global Nutrition Report: 20%), aligning with nutritional neuroscience documenting irreversible cognitive impacts at lower malnutrition intensities than acute physiological crises (16, 35). This contradicts health policy assumptions that educational concerns emerge only in emergency contexts, justifying nutrition-for-education programs for populations below health-sector radar.

#### 4.2.3 Spatial interdependencies confirmation

Our spatial clustering confirms and extends health spillover literature (58, 59): geographic adjacency dominance echoes HIV/AIDS geographic spread patterns (58) and governance spillovers through contiguous borders (59). However, nutrition-education spillovers exhibit stronger spatial dependence (*R*^2^ = 0.661 vs. 0.45–0.55 for health alone), suggesting educational systems amplify spatial transmission through cross-border student and teacher mobility and policy learning dynamics.

#### 4.2.4 Benefit-cost ratio validation

Estimates of 15:1 to 30:1 in critical-regime countries align with Hoddinott et al. (3)'s synthesis while exceeding conventional education investments–teacher training (3:1 to 8:1), infrastructure (2:1 to 5:1), learning materials (2:1 to 4:1) (51). Crucially, our regime-specific estimates reveal this advantage concentrates in critical-regime contexts (BCR 20:1 to 30:1) vs. moderate-regime settings (BCR 8:1 to 12:1), refining universal cost-effectiveness assumptions and providing precise geographic targeting guidance absent from aggregate estimates.

### 4.3 Hidden educational costs and policy reframing

The divergence between the educational impact threshold (4.37%) and health-sector emergency cutoffs–established in Sections 3.2.3 and 4.2— carries a direct policy implication: neurobiological mechanisms underlying educational impacts operate at lower malnutrition prevalence than acute physiological crises (16, 35, 61). Countries classified as “moderate burden” by WHO standards are already experiencing severe educational system impairment, justifying preventive programs (school feeding with micronutrient fortification, targeted supplementation, nutrition education) for populations overlooked by health systems focused on acute cases—and repositioning nutrition as core educational sector investment rather than auxiliary health expenditure.

### 4.4 Cost-effectiveness of geographic targeting

Policy simulations comparing a fixed USD 1.5 billion budget (approximately 0.1% of Sub-Saharan Africa GDP) across two strategies demonstrate critical resource allocation implications. Strategy A (universal): uniform allocation achieves 1.2 pp average malnutrition reduction and +1.46 pp LSCR gain. Strategy B (targeted): concentrating 80% on 27 critical-regime countries achieves 2.5 pp reduction (+4.62 pp LSCR in critical-regime countries; +0.8 pp reduction in moderate-regime countries) for a weighted gain of +2.85 pp—a 95% improvement in educational returns per dollar invested. Cost per LSCR point gained: USD 1.03 billion (universal) vs. USD 526 million (targeted).

Hierarchical targeting: Cross-country targeting represents the first tier of allocation. Within critical-regime countries, resources should be further targeted subnationally—for example, within Nigeria (national 8%), northern states (15–20%) receive intensive CMAM and universal school feeding while southern states (3–5%) receive preventive supplementation. Additional within-country targeting would further improve cost-effectiveness beyond the 95% gain, though data constraints limit immediate implementation in many countries (Section 4.10).

Reducing malnutrition by one percentage point in critical-regime countries yields estimated lifetime earnings gains of USD 2,500–3,000 per student cohort. Against intervention costs—school feeding (USD 50–80 per child annually), CMAM (USD 150–200 per treated child)—this generates benefit-cost ratios of 15:1 to 30:1, among the highest documented for any development intervention (3, 51).

### 4.5 SDG integration and strategic positioning

SDG 2 (Zero Hunger) and SDG 4 (Quality Education) are not parallel objectives but sequentially dependent: in critical-regime contexts (78% of countries), educational improvements achieve only 30–50% of potential returns when students' compromised cognitive capacities limit ability to benefit from improved provision (12, 13). This justifies integrated financing–nutrition investments framed as cross-cutting enablers advancing SDG 2, SDG 3, and SDG 4 simultaneously, with budgets allocated to “nutrition for human capital” rather than siloed by sector. Sub-Saharan Africa's youthful demographics—60% under age 25, adding 20–30 million annually to labor forces—amplify the urgency: human capital investments in schools and teachers will achieve suboptimal returns in 78% of countries until malnutrition is addressed, positioning nutrition as a prerequisite foundational investment for the continent's development trajectory.

### 4.6 Evidence-based intervention framework

The identified threshold provides actionable guidance for four differentiated intervention tiers:

#### 4.6.1 Tier 1—urgent intervention (PM > 40%)

Chad (39.9%), CAR (41.2%), Burundi (48.7%), Madagascar (42.0%), Niger (47.8%) require emergency responses: scaled CMAM with RUTF (75–85% cure rates), humanitarian food distributions, blanket supplementary feeding, integrated nutrition-health-WASH, and resilience building. *Investment: USD 75–150 per child annually. Expected LSCR gains: +20 to +33 pp over 7–10 years*.

#### 4.6.2 Tier 2—intensive programs (15% < PM ≤ 40%)

Eritrea, Ethiopia, Mozambique, Mali, Burkina Faso, Malawi require multisectoral approaches: CMAM for SAM cases, universal fortified school feeding, maternal-infant nutrition programs (antenatal supplementation, breastfeeding promotion, complementary feeding counseling), agricultural interventions (biofortification, homestead food production), integrated WASH, and nutrition-sensitive social protection. *Investment: USD 40–75 per child annually. Expected gains: +15 to +25 pp over 5–8 years*.

#### 4.6.3 Tier 3—preventive programs (4.37% < PM ≤ 15%)

Ghana, Kenya, Tanzania, Senegal, Côte d'Ivoire, Nigeria, Namibia, Zambia, Zimbabwe require preventive interventions: universal school feeding, targeted supplementation for at-risk groups (pregnant women, young children, adolescent girls), nutrition education, food fortification (salt iodization, flour and oil fortification), and surveillance systems detecting deterioration. *Investment: USD 20–40 per child annually. Expected gains: +3 to +8 pp*.

#### 4.6.4 Tier 4—maintenance (PM ≤ 4.37%)

Mauritius, Seychelles, Cape Verde, Botswana, South Africa, Rwanda, Gabon require targeted interventions for vulnerable subpopulations, micronutrient correction, fortification quality assurance, and double burden management. *Investment: USD 10–25 per child annually. Expected gains: +0.5 to +2 pp*.

#### 4.6.5 Within-country application

Countries straddling tier boundaries require differentiated subnational strategies using DHS, SMART surveys, or surveillance data–available in 18 of 35 countries but requiring data infrastructure investments in others. Cross-tier differentiation within countries amplifies the cost-effectiveness gains documented in Section 4.4.

### 4.7 Regional coordination mechanisms

Spatial clustering justifies regional nutrition policy coordination through ECOWAS, EAC, SADC, ECCAS, and IGAD through four priority mechanisms: (i) harmonizing intervention standards (school feeding specifications, CMAM protocols, supplementation guidelines, biofortification varieties) enabling 15–30% cost reductions through joint procurement; (ii) establishing Regional Emergency Nutrition Response Mechanisms with pre-positioned RUTF stockpiles, deployable nutrition personnel, and rapid-release financing; (iii) facilitating South-South knowledge exchange through learning platforms, staff secondments, and regional centers of excellence; and (iv) coordinating advocacy and resource mobilization from international partners.

Implementation requires addressing three dimensions. Multisectoral coordination: National Nutrition-Education Councils integrating Ministries of Health, Education, Agriculture, Finance, and Social Protection develop unified action plans with education-sector targets. Financing: Blend domestic mobilization (education budget reallocation, earmarked taxes, agricultural subsidy reform) with international financing (Global Financing Facility, Education Cannot Wait, bilateral ODA) and private engagement (public-private partnerships, impact bonds). Monitoring: Link nutrition surveillance with education management information systems, establishing 4.37% as the threshold triggering intensified interventions.

### 4.8 Methodological contributions and innovations

This study introduces a hybrid PTR-GNN framework—to our knowledge the first application to nutrition-education dynamics—enabling simultaneous identification of non-linear regime shifts and spatial interdependencies while maintaining analytical interpretability. As detailed in Sections 2.2–2.4, PTR provides regime-specific conditional associations for threshold identification and policy targeting (validated by Pesaran CD test, *p* = 0.061), while GNN captures spatial interdependencies for regional coordination and prediction (*R*^2^ = 0.661). This overcomes the twin limitations of conventional panel methods–imposed linearity and spatial independence–without sacrificing interpretability. The framework provides a replicable template for studying threshold non-linearities and spatial clustering in other human development domains: health-education linkages, poverty-conflict dynamics, and climate-agriculture relationships.

### 4.9 Methodological note: climate, conflict, and price variables

As established in Section 2.1, climate vulnerability, conflict intensity, and price volatility are *mediators* rather than confounders: drought → malnutrition → cognitive impairment → reduced attainment; conflict → nutritional status → learning capacity. Controlling them would absorb the malnutrition association of interest, yielding downward-biased estimates irrelevant for policy (52, 53). PTR estimates total conditional associations while GNN captures spatial dependencies from these shared vulnerabilities via geographic adjacency—avoiding both mediator bias and omitted variable bias. Robustness confirms results are not artifacts of these omissions: threshold stability across 1990–2024 windows (0.08 pp variation), geographic holdout consistency (*R*^2^ = 0.59–0.70), and null placebo tests validate the approach (Section 3.4). Future mediation analysis could decompose climate vs. conflict pathways; for current policy purposes, total association estimates are the appropriate estimand.

The individualistic fallacy—assuming micro-level effects generalize linearly to macro returns—is as problematic as the *ecological fallacy*. Nutrition policy has suffered more from the former: school feeding raising individual attendance 5% does not imply national programs raise aggregate completion proportionally, because general equilibrium effects—peer dynamics, teacher responses, labor market adjustments, fiscal feedbacks—only emerge at population scale. Our regime-specific estimates (β_1_ = −0.31, β_2_ = −1.85) capture total equilibrium associations for national resource allocation; district- and household-level analyses provide complementary subnational guidance but cannot substitute for macro-level estimates when informing continental policy priorities.

### 4.10 Limitations and future research directions

Several limitations warrant acknowledgment and suggest productive research directions:

#### 4.10.1 Macro-level aggregation

National-level analysis masks within-country heterogeneities, raising ecological fallacy concerns. However, our objective is explicitly macro: micro treatment effects do not generalize linearly due to general equilibrium mechanisms observable only at population scale; threshold associations (4.37%) represent emergent system properties identifiable only through cross-country variation; and national budgets require system-wide comparisons. The 22%/78% regime imbalance reflects the genuine malnutrition distribution–not a sampling artifact—with bootstrapped β_1_ CIs ([−0.412, −0.213]) and regional subsample analyses confirming precise estimation (Section 3.4, Table 4 Panel F). District-level analysis would provide complementary subnational targeting: preliminary DHS evidence suggests urban thresholds (3.5–4.0%) differ from rural (5.0–5.5%), and data availability enables comprehensive subnational targeting in only 18 of 35 countries–expanding surveillance systems is a priority. Cross-country targeting (Section 4.4) represents the first tier; within-country geographic and household targeting would further amplify cost-effectiveness.

#### 4.10.2 Causal identification

Reverse causality remains a substantive concern: education may reduce malnutrition through income growth and health knowledge channels. While neurobiological directionality—malnutrition during the first 1,000 days preceding school entry—provides strong *a priori* grounds, simultaneous feedback cannot be fully excluded. Future research should exploit exogenous instruments: international food price shocks, drought-induced agricultural crises, and mass micronutrient supplementation campaigns. Regression discontinuity designs around CMAM program launches could provide locally valid estimates. Until then, β_1_ = −0.312 and β_2_ = −1.847 should be interpreted as robust conditional associations rather than strictly causal effects.

#### 4.10.3 Omitted variable considerations

Residual time-varying confounding from health infrastructure, sanitation, conflict, and climate may persist beyond country fixed effects. As detailed in Section 4.9, health and sanitation are mediators rather than confounders; conflict and climate are captured via GNN geographic adjacency. Threshold estimates remain within 0.23 pp across all specifications (amplification 5.6:1 to 6.3:1), and imputation sensitivity analyses yield estimates within 0.08 pp of baseline (Table 4, Panels B, C, F). Future research incorporating time-varying conflict and climate indices—using formal mediation analysis (52, 53)—would strengthen identification; panel vector autoregression could test temporal ordering between malnutrition, conflict, climate, and educational outcomes.

#### 4.10.4 Spatial dependence in PTR

PTR treats countries as independent units, validated by Pesaran's CD test (*p* = 0.061; Section 2.2). Future research could explore spatially-augmented threshold regression—incorporating spatial lags of malnutrition or LSCR–using Driscoll-Kraay standard errors (62) with Hansen's threshold detection, or spatial quantile threshold regression to examine whether associations vary across the LSCR distribution.

#### 4.10.5 Aggregate indicator constraints

Underweight does not distinguish stunting vs. wasting pathway contributions, nor identify specific micronutrient deficiencies with distinct neurobiological thresholds. While stunting ($\hat{\gamma }=28.5\%$, 4.8:1) and wasting ($\hat{\gamma }=3.91\%$, 5.9:1) confirm robustness (Section 2.1), future research linking anthropometry with biomarker data (hemoglobin, serum ferritin, urinary iodine) could examine threshold heterogeneity across deficiency types. Composite indices integrating stunting, wasting, and micronutrient status could reveal finer-grained policy red lines below 4.37%.

#### 4.10.6 Timing ambiguity

Current analysis cannot distinguish deficit timing (prenatal, 0–24 months, preschool, school-age), yet timing criticality is well-established (15, 16). Longitudinal cohort studies linking prenatal biomarkers to educational trajectories could reveal lower thresholds for early infant periods when neuroplasticity peaks.

#### 4.10.7 Long-term outcomes

LSCR captures immediate attainment but not learning quality, labor market outcomes, or intergenerational transmission. Following cohorts into adulthood would quantify full lifetime returns, likely revealing higher benefit-cost ratios as cognitive benefits compound (9, 29).

### 4.11 Concluding remarks

This study demonstrates child malnutrition represents not merely a health imperative but a strategic lever for educational transformation in Sub-Saharan Africa. Panel Threshold Regression identifies a critical threshold at 4.37%—substantially below health emergency cutoffs—where malnutrition's educational associations intensify six-fold (β_2_ = −1.85 vs. β_1_ = −0.31), consistent with exhausted compensatory mechanisms. With 78% of analyzed countries operating above this threshold, malnutrition constitutes a binding structural constraint on human capital formation across the continent. Graph Neural Network analysis identifies three high-vulnerability regional clusters (Sahel, Central Africa, East Africa Horn) with shared nutritional drivers justifying coordinated responses through existing integration frameworks (ECOWAS, ECCAS, IGAD).

Policy simulations demonstrate concentrating resources on critical-regime countries achieves 95% greater educational returns than uniform allocation, with benefit-cost ratios (15:1 to 30:1) exceeding conventional education investments (3:1 to 8:1). The policy message is unambiguous: achieving SDG 4 (Quality Education) requires first addressing SDG 2 (Zero Hunger). For Sub-Saharan Africa's 400 million children whose cognitive development during the first 1,000 days determines lifetime learning capacity and economic productivity, and for the continent's future prosperity which depends critically on human capital quality of youthful populations entering labor markets, there is no more important, urgent, or higher-return investment than ensuring adequate nutrition. The empirical evidence is clear, the policy frameworks are defined, the interventions are proven effective, and the returns are compelling. What remains is action.

## Data Availability

The original contributions presented in the study are included in the article/supplementary material, further inquiries can be directed to the corresponding author.

## Footnotes

1 AdamW optimizer with learning rate 5 × 10^−4^; dropout (0.1); gradient clipping (threshold = 1.0); early stopping (patience = 25 epochs); temporal cross-validation using rolling windows. Training converges at epoch 73/100, training-validation loss gap below 5%.
